# Supplementary material for: Electrically-driven Yagi-Uda antennas for light
Source: Nat Commun. 2020 Jan 8;11:115. doi: 10.1038/s41467-019-14011-6 (PMC6949256; doi:10.1038/s41467-019-14011-6)
Supplement: Supplementary file 1 — Supplementary Information [file 41467_2019_14011_MOESM1_ESM.pdf]

SUPPLEMENTARY INFORMATION

Electrically-Driven Yagi-Uda Antennas for Light

Kullock et al.

Supplementary Note 1: Modelling of Yagi-Uda Antennas

In the simplest case a Yagi-Uda antenna consists of one reflector, a feed element and directors as sketched in supplementary figure 1. To achieve phase-coherent emission of all antenna elements in one direction, the resonance frequency of the reflector is detuned to longer wavelengths, while the resonance frequency of the directors is detuned to shorter wavelengths in relation to the driving frequency of the feed element. This leads to phase differences between the individual elements and results in constructive interference in forward as well as destructive interference in backward direction. To achieve a high forward-to-backward (FB) ratio the geometric parameters of the antenna structures need to be optimized. An analytical model is used for a quick overview analysis and more realistic structures are then simulated using the boundary element method (BEM) as well as finite-difference time-domain (FDTD) simulations.

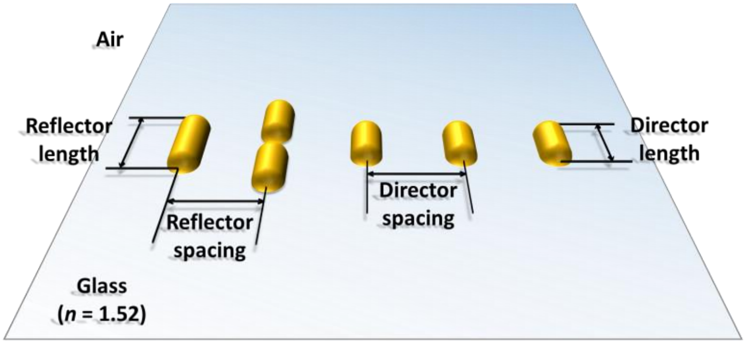

Supplementary Figure 1. Sketch of a Yagi-Uda antenna consisting of one reflector, a feed element and three directors.

Supplementary Note 2: Forward-to-backward Ratio

The FB ratio is determined from simulated and measured far-field emission patterns (as shown in Fig. 3 in the main text) in two different ways.

The first is the 'pixel' method used by Curto et *al.*[1] where the most intense pixel in forward direction is selected and compared to the diametrical opposite pixel on the emission pattern. For both pixels the background (CCD offset) is subtracted and then the forward pixel is divided by backward one to obtain the ratio. This method provides straight-forward results and is well established in literature, however, it is also very prone to errors as noise in backward direction can lead to high FB-ratio fluctuations. This is reduced for high signal-to-noise ratios and for our experimental data we estimate the error to be ±1.4 dB.

The second way is the 'areal' method introduced by Gurunarayanan et *al.*[2]. By integrating the emission pattern over a solid angle section in forward and backward direction the fluctuations are greatly reduced. However, as the intensity drops when moving away from the forward maximum the value is not fixed and decreases with increasing ($\Delta\theta$, $\Delta\varphi$). Hence, it is inherently smaller than the one of the 'pixel' method and based on the uncertainty of the emission pattern size we estimate the error to be ±0.2 dB.

Our compromise for the FB ratio is to state both values in the main text while using the same intervals as Gurunarayanan ($\Delta\theta$ = 15°, $\Delta\varphi$ = 20°) to better compare the results to the literature. The used solid-angle intervals are indicated in Fig. 3i (main text).

Supplementary Note 3: Analytical Model

To quickly estimate resonance frequencies and spacings of the passive antenna elements a dipole interaction model is employed[3]. We consider the individual particles to be much smaller than the wavelength such that the field can be assumed to be homogenous over each particle. In this quasi-static approximation all antenna elements are treated as coupled dipoles as pictured in supplementary figure 2. The local electric field at a dipole E_{loc} is given by the emitted fields of the feed element (E_{feed}) and of the other dipoles (E_{j}):

$$E_{\mathrm{loc,i}} = E_{\mathrm{feed}} + \sum_{\mathrm{j}\neq\mathrm{i}} E_{\mathrm{j}}$$

The quasi-static polarizability $\alpha(\omega)$ of metallic nanoparticles is modeled[4] as a function of effective particle volume V_{eff}, dielectric function ϵ_{NP}, and the depolarization factor N[5] which describes the geometry of prolate spheroids,

$$\alpha(\omega) \propto \frac{V_{\mathrm{eff}}}{\frac{1}{\left(\frac{\epsilon_{\mathrm{NP}}}{\epsilon_{\mathrm{surr}}} - 1\right)} + N - i(V_{\mathrm{eff}}\omega^3/6\pi c^3)}$$

with ϵ_{surr} the dielectric constant of the surrounding and ω the light frequency. The total electric field due to an induced dipole can then be written as

$$E_{\mathrm{j}} = \frac{k^3}{\mathrm{i}} \alpha_{\mathrm{j}} E_{\mathrm{loc,j}} \frac{e^{\mathrm{i}kr_{\mathrm{j}}}}{(kr_{\mathrm{j}})^3}\left[\mathrm{i}(kr_{\mathrm{j}})^2 - kr_{\mathrm{j}} - \mathrm{i}\right] = A_{\mathrm{j}} \frac{e^{\mathrm{i}kr_{\mathrm{j}}}}{(kr_{\mathrm{j}})^3}\left[\mathrm{i}(kr_{\mathrm{j}})^2 - kr_{\mathrm{j}} - \mathrm{i}\right]$$

where we introduce the complex dipole amplitude $A_{\mathrm{j}} = -\mathrm{i}k^3 \alpha_{\mathrm{j}} E_{\mathrm{loc,j}}$. $k = \frac{\omega}{c}$ is the wavenumber and r_{j} denotes the distance between j-th dipole position and observer on the dipole-chain axis (x-axis in supplementary figure 2). We express the amplitude of the j-th dipole by the shorthand notation

$$A_{\mathrm{j}} = \frac{E_{\mathrm{loc,j}}}{1 + \gamma_{\mathrm{j}} + \mathrm{i}\delta_{\mathrm{j}}}.$$

Here, γ_{j} describes absorption losses and δ_{j} is the effective resonance detuning of the j-th dipole compared to the driving frequency ω_0 of the feed. The detuning can be adjusted via the depolarization factor N, i.e. the geometry and dielectric function of the particle. Thus, we obtain an equation system for the dipole amplitudes whose solution is found in a self-consistent way for all antenna elements. This leads to the far-field emission pattern in the plane perpendicular to the dipole axes[3] (xz-plane in supplementary figure 2)

$$I(\theta)\big/I_{\mathrm{F}} = \left|1 + \sum_{\mathrm{i}} \frac{A_{\mathrm{i}}}{A_{\mathrm{F}}} e^{\mathrm{i}kr_{\mathrm{i}}\cos(\theta)}\right|^2.$$

It is normalized to the emission intensity of the feed I_{F} with the oscillation amplitude A_{F}. θ is the angle between antenna axis and the considered direction in the xz-plane and r_{i} is the distance between feed and i-th dipole (labeled as reflector/director spacings in supplementary figure 2). An example emission pattern for the values $\gamma_{\mathrm{ref}} = \gamma_{\mathrm{dir}} = 0.4$, $\delta_{\mathrm{ref}} = -0.4$, $\delta_{\mathrm{dir}} = 2$, reflector spacing 0.22λ, and director spacing 0.32λ

is depicted in Fig. 1c (main text). For a driving wavelength of λ_0 = 800 nm and ε_{surr} = 1.52, those detuning values correspond to a reflector resonance at 815 nm and director resonance at 740 nm for spheroidal particles.

The FB-ratio can now be optimized by adjusting the effective detuning (by varying the aspect ratio) of the antenna elements and the distances between reflector and feed as well as feed and director (see further down).

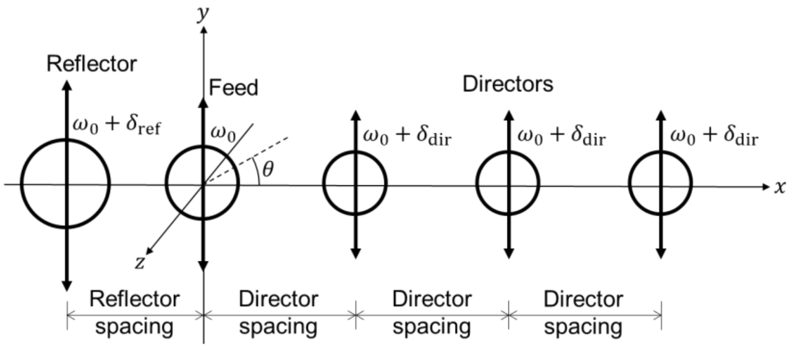

Supplementary Figure 2. Analytic coupled dipole model. All antenna elements are treated as dipoles in the quasi-static approximation and interact with each other. The driving frequency ω_0 of the feed is fixed, while reflector and director are detuned by δ_{ref} and δ_{dir}, respectively. The directors are equally spaced along the array.

Even though this method provides a quick and simple assessment that helps to understand the physics of Yagi-Uda antennas, it is limited by the quasi-static approximation, which is only valid for small particles, and the required homogeneous surrounding. Hence, additional full numerical models are necessary to better match the experimental conditions.

Supplementary Note 4: Numerical Models

The Boundary Element Method (BEM) is a frequency-domain algorithm to solve partial differential equations. By reducing the problem to the surface boundaries of materials, computational power and time can be saved compared to volume methods. In this work we used the Open-Source MNPBEM toolbox[6–8] which was developed to solve Maxwell's equations for metal nanoparticles in dielectric environments.

The Yagi-Uda antennas and the individual antennas were modeled using rounded polygons that are extruded via custom-made edge profiles as depicted in supplementary figure 3a. As the dielectric function of gold the data from Olmon et *al.*[9] were used, air (n = 1) in the top half-space and glass (n = 1.52) in the bottom were considered as well as retardation effects beyond the quasi-static approximation. The BEM model was used for optimizing the dimensions of the Yagi-Uda antennas, but also to study the influence of a gold nanoparticle in antenna gaps to predict and verify spectral shifts observed in DEP experiments. In the latter case the gold nanoparticle was placed into the gap of the two-arm antenna and scattering spectra were simulated upon plane wave illumination.

Unfortunately, BEM cannot handle too elongated/complex shapes such as the kinked connectors of the feed elements as they lead to ill-defined matrixes and unstable numerical results.

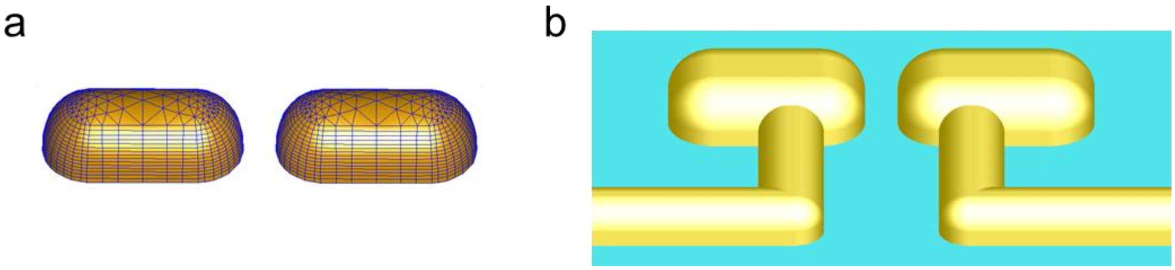

Supplementary Figure 3. (a) BEM geometry of a two-arm nanoantenna. Its surface is discretized into triangles and quadrilaterals. (b) FDTD geometry (rendered) of an antenna structure with connectors on a glass substrate. All top edges of metallic structures are rounded.

Therefore, the finite-difference time-domain (FDTD) method[10] was used for modelling the influence of the connectors (FDTD solutions version 8.16, Lumerical Solutions Inc, Vancouver, CA). In contrast to BEM, FDTD discretizes the whole volume of a structure as well as time itself. Therefore, the particle shape can only be approximated via (small) cubic building blocks, which lead to "staircasing" effects, and the open boundary conditions need to be emulated with perfectly matched layers (PML)[11]. In our model the connectors pass through the PML and are thus treated as infinitely long metal wires without reflections – c.f. supplementary figure 3b. The optimal position of the connectors is discussed below.

Supplementary Note 5: Position of the Connector

To drive Yagi-Uda antennas electrically metallic contact wires are necessary to connect the feed element to an external voltage source. Due to the significant size of wires in the nanoworld, the connector attachment position as well as their routing must be optimized in order to not disturb the operation of the feed and the passive elements. Additionally, it must be possible to fabricate the structure using the FIB milling technique (see supplementary notes 7 and 8).

FDTD simulations have been performed to find the optimal attachment position and routing. Dipole antennas were modeled with an adaptive mesh size down to $(1\text{nm})^3$ and a Gaussian source, polarized parallel to the antenna long axis, was chosen to excite the structure through the glass substrate (n = 1.52) at normal incidence. The dipole antennas were then contacted at different positions. First at the end of the respective arms pointing straight away from antenna and then in the middle of the of the arms – similar to the connected dipole antennas in[12] but this time both connectors were mounted on the same side and featured a kink in order to accommodate the reflector left to the feed element. Sketches and results are depicted in supplementary figure 4.

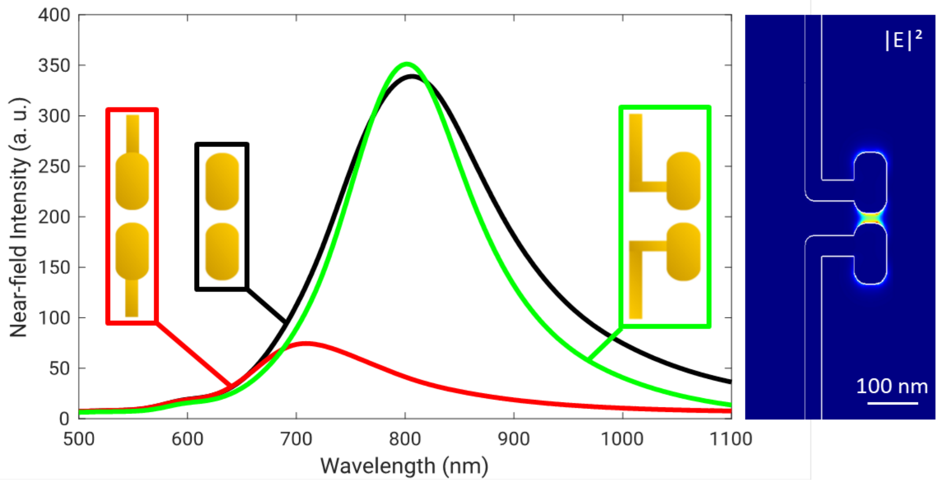

Supplementary Figure 4. Influence of the connectors. Left: FDTD simulated near-field intensities for different connector configurations. If the connectors are attached from the top and bottom (red) the near-field intensity of the antenna is strongly decreased while the one-sided configuration (green) conserves the resonance and amplitude of the antenna as compared to the case without connectors (black). Right: Near-field intensity map for the chosen one-sided configuration.

As one can see, analogous to Prangsma et *al.*[13], wires attached at positions of high field intensity, i.e. the ends of the antenna arms (red), strongly decrease the near-field intensity as compared to an antenna without any connectors (black). However, by attaching the wires from the side at positions of low field intensities (green), the performance of the antenna is nearly unaltered. The associated near-field intensity map of this configuration shows high confinement of electrical fields in the antenna gap and no significant near-fields along the connectors, i.e. the energy leakage is minimal. For those reasons, we were able to ignore the connector wires when optimizing the dimensions of parasitic elements using BEM.

Note, that the kinked connectors can also positively contribute to a directed emission. Supplementary figure 5 shows SEM images and experimental/simulated emission patterns of a regular dipole antenna as well as a feed antenna connected from the left-hand side are depicted. The regular dipole antenna shows basically no directionality which is expected due to symmetry consideration. However, for the feed antenna FB ratios exceeding 2 dB can be observed. This already is a remarkable directionality and the reason for this effect is that the parts of the connector that run parallel to the feed act as reflecting elements. This leads to an emission to the right which can further be optimized by tuning the distance between antenna and the kink in the connecting wires. However, in our Yagi-Uda antennas we are restricted by the available space to the reflector such that we had to set the connector spacing to 100 nm. Nevertheless, this effect helps to improve the performance of the Yagi-Uda antennas especially for low numbers of directors.

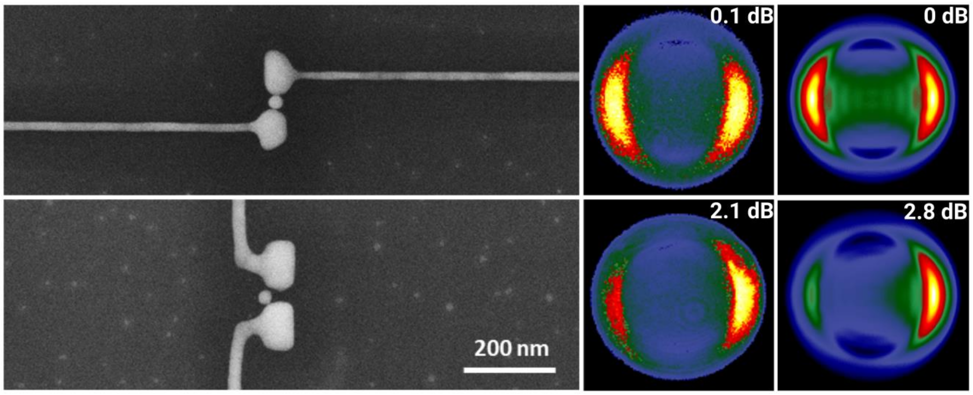

Supplementary Figure 5. SEM micrographs (left) of a dipole and a feed antenna with kinked connectors. Measured (middle) and simulated (right) electroluminescence emission patterns are in very good agreement. The FB ratios are calculated by integrating over solid angle sections, as depicted in Fig. 3i in the main text. The kinked connectors already lead to directional emission with a FB ratio exceeding 2dB.

Supplementary Note 6: Optimal Geometry

For optimizing the geometry of the Yagi-Uda antenna (reflector/director wavelength and spacing) we used the FB ratio as figure of merit. Since the feed resonance shifts upon placing a particle into the gap by means of dielectrophoresis, the “spectral window” between reflector and director resonances has to be sufficiently broad to reproducibly fabricate stable forward emitting antennas. Therefore, we optimize the director spacing for fixed reflector resonance at 890 nm and director resonance at 680 nm. The connector wires of the feed element and general fabrication limitations required a fixed reflector spacing of 200 nm ≈ $\lambda/4$.

In order to calculate the absorption parameters γ_{ref} and γ_{dir} required for the coupled dipole model, the scattering spectra displayed in Fig. 2j (main text) were fitted using the polarizability $\alpha(\omega)$ as stated above and an effective homogeneous background medium with $\epsilon_{\mathrm{surr}} = 1.52$. With the resulting effective volumes V_{eff} from the fit we obtain $\gamma_{\mathrm{ref}} = 0.370$ and $\gamma_{\mathrm{dir}} = 0.531$. A subsequent map of the FB ratio depending on the director spacing and driving wavelength is depicted in supplementary figure 6a. As one can see the FB ratio reaches a maximum for a wavelength of ~860 nm and spacings of 330 nm, 200 nm and below 100 nm.

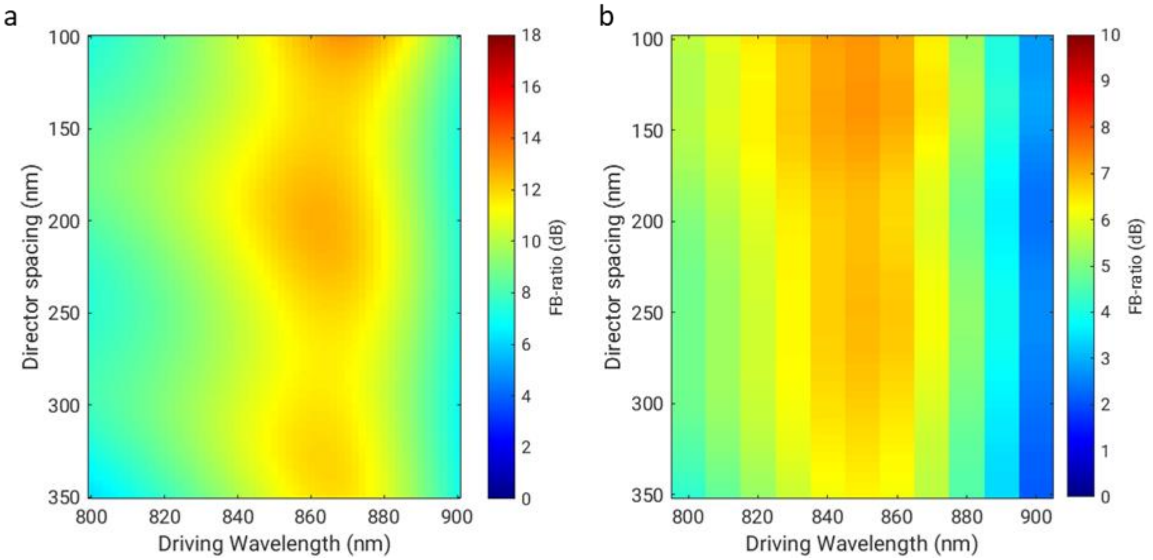

Supplementary Figure 6. Director spacing optimization maps for a Yagi-Uda antenna with one reflector, feed element, and three directors. Results for the coupled dipole model and the BEM algorithm are shown in (a) and (b), respectively.

For similar but more realistic FB maps with the BEM model, a connector-less Yagi-Uda antenna was assumed with the same reflector/director resonance wavelengths and reflector spacing as mentioned above. A dipole source polarized parallel to the long axis of the antenna was placed inside the feed gap centrally 15 nm above the substrate. The driving wavelength of the dipole was swept while the geometry of the feed element itself was kept constant. The results are plotted in supplementary figure 6b where one can see that the optimum driving wavelength blue-shifted slightly to ~850 nm and the director spacings to values of ~240 nm and ~130 nm. Furthermore, the FB ratio decreased noticeably – probably due to retardation effects and the asymmetric dielectric surrounding. Nevertheless, director spacings around 240 nm and driving wavelengths ~850 nm seem to be a good starting point for the experiments.

Supplementary table 1 lists an overview of all the obtained parameters from the calculations and experiments.

**Parameter**		**Quasi-Static**	**BEM**	**FDTD**	**Experiment**
Connector	*Width*	-	-	30 nm	30 nm
	Spacing	-	-	100 nm	100 nm
Reflector	*Length*	-	180 nm	166 nm	166 nm
	Height	-	40 nm	40 nm	40 nm
	Width	-	60 nm	60 nm	60 nm
	Spacing	200 nm	200 nm	200 nm	200 nm
Feed	*Length*	-	145 nm	145 nm	115 nm
	Height	-	40 nm	40 nm	40 nm
	Width	-	80 nm	80 nm	80 nm
	Gap	-	30 nm	30 nm	25-30 nm
Director	*Length*	-	111 nm	108 nm	108 nm
	Height	-	40 nm	40 nm	40 nm
	Width	-	60 nm	60 nm	60 nm
	Spacing	330 nm	240 nm	240 nm	240 nm
Particle	*Diameter*	-	-	-	30 nm

Supplementary Table 1. Synopsis of optimum geometric parameters and dimensions of fabricated antennas.

Supplementary Note 7: General Sample Layout and Fabrication

Microscope cover slips (#1, Gerhard Menzel GmbH, Saarbrücken, DE) are used as substrates with evaporated Au/Cr electrodes of 70nm/5nm thickness[12]. Cover glasses contain three electrode areas, each having six top, six bottom and one ground contact. Single-crystalline gold flakes are grown on separate glass substrates[12,14,15], individual flakes are selected and covered with droplets of Polymethylmethacrylate (PMMA). The substrates are tempered for 1 hour at 160 °C to harden the PMMA and afterwards a micromanipulator needle is used to transfer individual droplets to the electrode areas. After further drying the droplets for 5 hours at 100 °C, the PMMA is removed by rinsing the sample with acetone to expose the bare gold flakes contacting the electrodes. Afterwards, the single-crystalline gold flakes are structured using focused-ion beam milling (FIB, Helios Nanolab 600)[16]. In supplementary figure 7 the resulting structure layout is depicted. By using a sourcemeter (Keithley 2636B, Tektronix Inc., Beaverton, USA) and contacting the electrode structures with micromanipulator needles a voltage can now be applied over individual 20-nm antenna gaps.

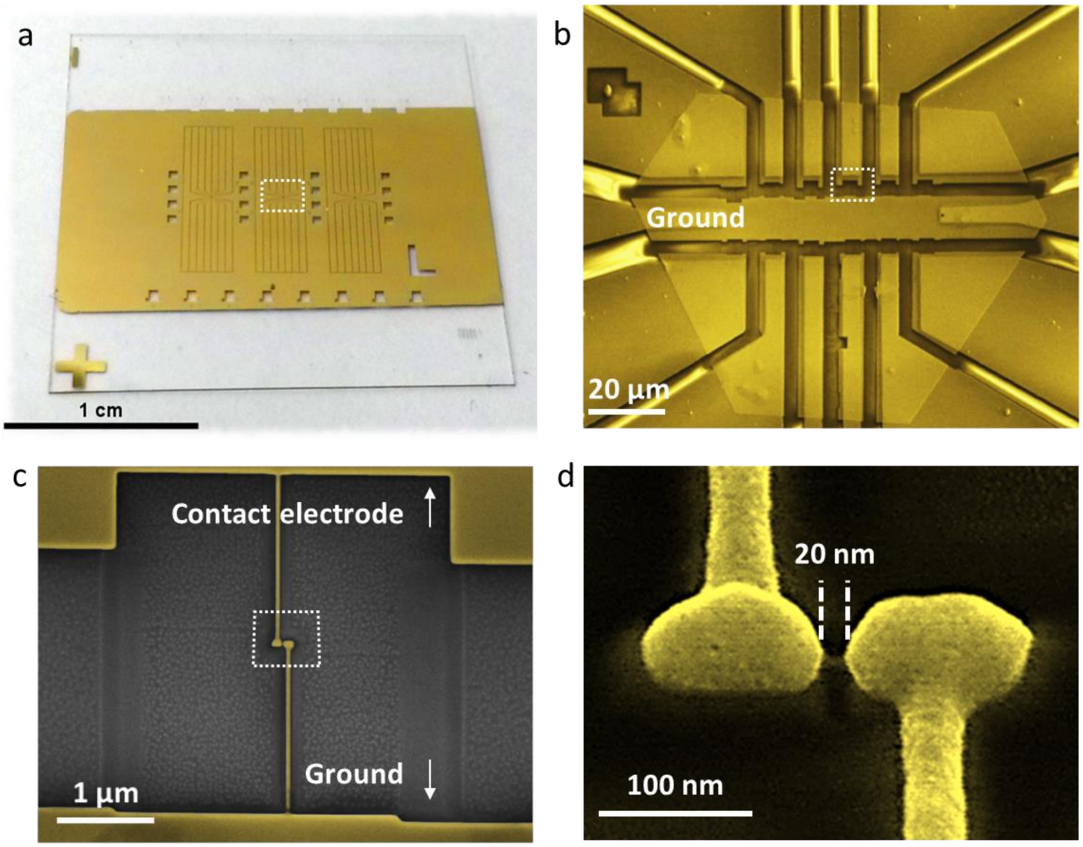

Supplementary Figure 7. Structure layout. (a) Microscope glass cover slip with three areas of evaporated Au/Cr electrode structures. (b) SEM image of a gold flake that was transferred onto the electrode structures after focused ion beam milling. The ground electrode is indicated. (c) Zoom-in to an antenna structure including connectors and electrodes. (d) Further zoom-in to the antenna featuring a gap of 20 nm.

Supplementary Note 8: Advanced Focused Ion Beam Milling and Etching

High-precision FIB milling of single-crystalline gold flakes is required for fabricating accurate electrically-connected gold nanostructures such as optical antennas[12,13]. More complex systems such as optical Yagi-Uda antennas are even more demanding as the connector wires need to be kinked and additional passive elements must be accurately structured and precisely positioned in close proximity. By using existing processes only unsatisfying results can be obtained (see supplementary figure 8b) and, hence, we had to improve the fabrication in several aspects.

Firstly, as thinner flakes allow narrower FIB cuts, we optimized the flake growing process to obtain very thin (30-50 nm) but still laterally large-enough flakes[14] and developed the above described transfer process to still place them flawlessly on the electrode structures.

Secondly, to reduce the charge-induced drift while FIB milling, we extended the contacting electrodes reaching far to the outside (c.f. supplementary figure 7a) and constructed a custom-made sample holder to properly ground all of them while FIB milling.

Thirdly, we improved the milling procedure. So far, the fabrication of nanoantennas was performed by arranging several rectangles as shown in supplementary figure 8a and milling them in the following order:

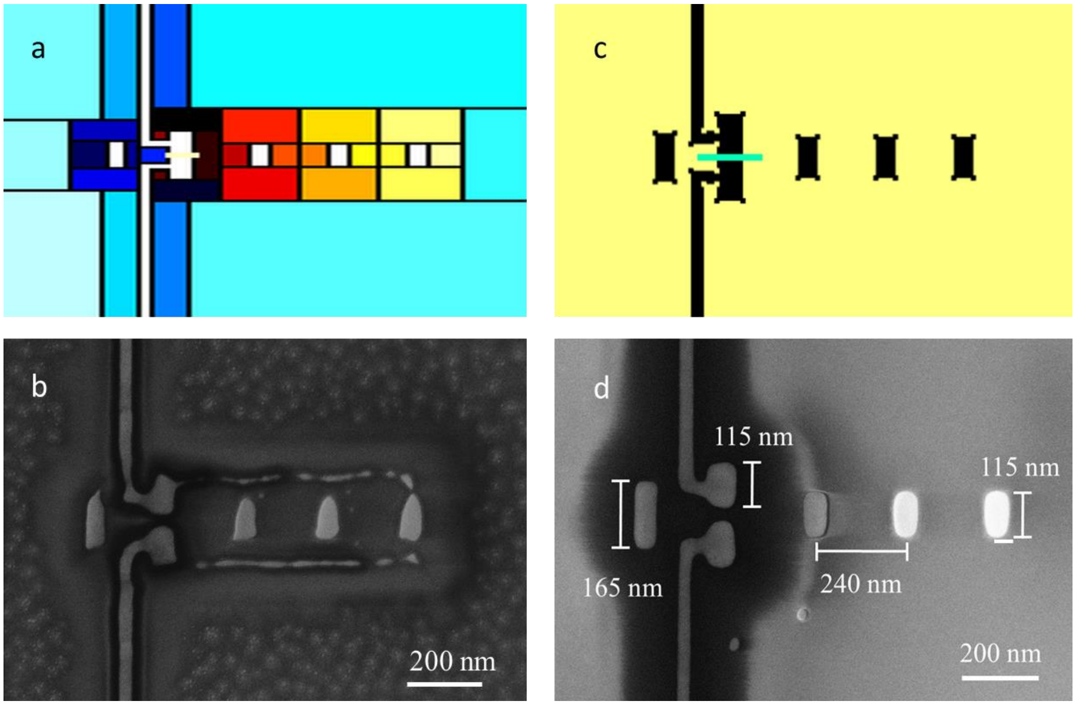

Supplementary Figure 8. Comparison between traditional and enhanced milling. (a) Traditional FIB layout for a Yagi-Uda antenna based on rectangles. (b) Resulting Yagi-Uda antenna showing a top-bottom asymmetry, a poor quality of the kinked connectors and a residual gold wall around the directors. (c) Bitmap used for the enhance milling. The yellow area is milled from right to left and the gap is made in a separate step, indicated by the cyan line. Extra pixels at the corners of structures account for proximity effects. (d) Resulting Yagi-Uda antenna fabricated with the bitmap and the enhancements mentioned in the text. The structures are symmetric, the kinks are well proportioned anc no residual gold can be found.

First, all light-blue rectangles are milled in parallel. This is the time-consuming removal of the vast majority of the gold and done separately to minimize drift effects. Then the high-precision structuring of the antennas is performed by quickly parallel-milling the colored rectangles. Finally, the gap is cut using a single line. This technique works well for connected dipole antennas, however, for optical Yagi-Uda antennas redeposition and charging effects are a major hurdle: As displayed in supplementary figure 8b the resulting shape of the individual elements is not smooth and a gold wall remains around the directors affecting the antenna performance.

For this reason and due to the reduced drift with the custom-made sample holder, we implemented another milling technique based on bitmap files. Supplementary figure 8c depicts such a bitmap for Yagi-Uda antennas where a whole structure is milled in parallel based on the RGB color code of the pixels. Extra pixels at corners are added/omitted to minimize proximity effects and to obtain sharper features such as the kinked connectors. The gap is also milled separately by using a single line cut at the end. Supplementary figure 8d shows a resulting Yagi-Uda antenna that is very symmetric and no residual gold wall can be found.

One remaining issue with the focused-ion beam milling is that while structuring the gold is redeposited on the sample and forms small clusters. Supplementary figure 9a shows such clusters located around the elements of a freshly milled Yagi-Uda antenna. Especially inside the gap (inset) these clusters are critical as they strongly respond to electromagnetic fields, cause leakage currents and agglomeration to bigger gold particles, and thus disturb the electroluminescence measurements as well as the dielectrophoresis.

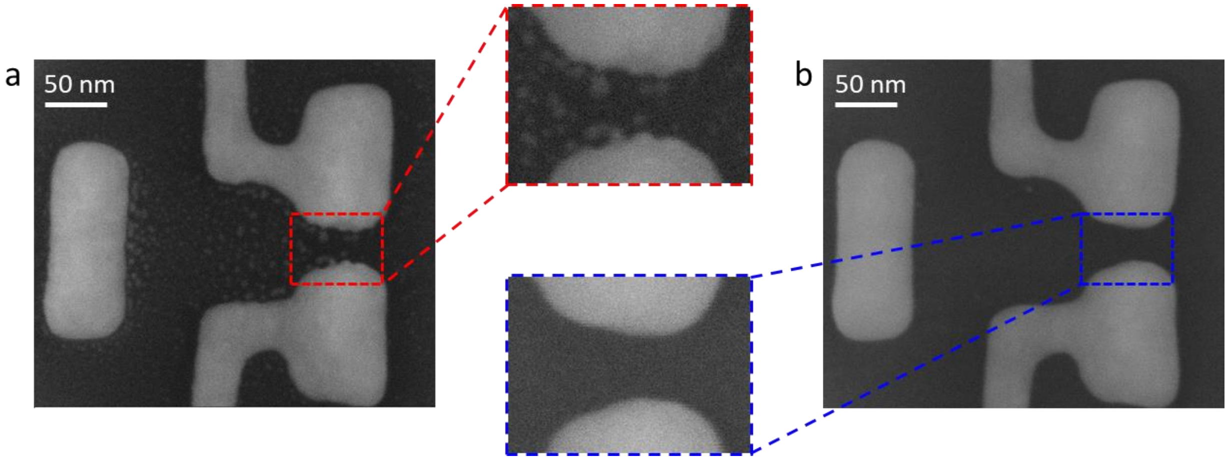

Supplementary Figure 9. SEM images of an antenna structure (a) before and (b) after KI etching. The dashed red (blue) square provides zoom-ins into the gap region before (after) etching.

To clean the gaps a home-made etching solution of I_2 : KI : H_2O = 1 mg : 4 mg : 40 ml is applied to remove the clusters and after 15-25 s washed away with purified water and ethanol. Additional etching steps can be added, but typically one etching step of 20 s duration is sufficient. Supplementary figure 9b shows a cleaned antenna structure with all clusters in the gap being removed. Note that the edges/corners of the antennas are smoothed by the etching process and the gaps are slightly enlarged, too.

Supplementary Note 9: Dielectrophoresis

An important part of the electrically-driven Yagi-Uda antennas is their gap, as it determines the physical properties of the structure and it facilitates the light generation. Because 1 nm gaps – necessary for inelastic electron tunneling – cannot be milled with FIB, we fabricated 25-30 nm gaps and further reduced their size by inserting 30 nm particles coated with a 1 nm organic shell.

In previous work atomic force microscope (AFM) pushing was used to insert particles into the gap of electrically-driven nanoantennas[12]. This method worked to fabricate proof-of-principle structures but has a low yield and is difficult to apply to more complex antenna geometries such as the Yagi-Uda design. Therefore, dielectrophoresis (DEP) exploiting the highly localized and enhanced fields in the antenna gap is performed to replace AFM pushing, and a feedback system based on white-light dark-field scattering is implemented to ensure that only single particles are trapped.

The key feature of DEP is that if one places a polarizable particle in a non-uniform electric field, the net force acting on it is not zero. Considering a homogeneous sphere with Ohmic losses, the time-average DEP force can be written as[17]

$$\langle F_{\mathrm{DEP}} \rangle = 2\pi\varepsilon_1 R^3 \left[\frac{\varepsilon_2 - \varepsilon_1}{\varepsilon_2 + 2\varepsilon_1} + \frac{3(\varepsilon_1\sigma_2 - \varepsilon_2\sigma_1)}{\tau_{\mathrm{MW}}(\sigma_2 + 2\sigma_1)^2(1+\omega^2\tau_{\mathrm{MW}}^2)}\right]\nabla E_{\mathrm{rms}}^2,$$

where ε_1 (ε_2) and σ_1 (σ_2) are the dielectric function and the conductivity of the suspending medium (particle), respectively. R is the radius of the sphere, ω is the frequency and E_{rms} is the root-mean-square magnitude of the electric field. $\tau_{\mathrm{MW}} = \frac{\varepsilon_2+\varepsilon_1}{\sigma_2+2\sigma_1}$ is called the Maxwell-Wagner charge relaxation time and describes the decay of dipolar distribution of free charge carriers at the sphere surface. In this work, gold nanoparticles are dissolved in water and one obtains a force depending $\omega\tau_{\mathrm{MW}}$ as shown in supplementary figure 10. The force is positive for small frequencies and becomes negative as $\omega\tau_{\mathrm{MW}}$ exceeds one. As

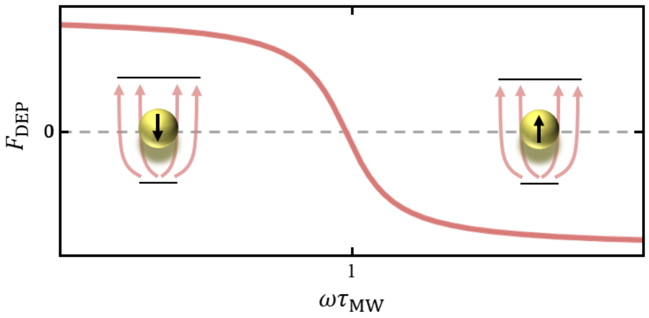

Supplementary Figure 10. F_{DEP} as a function $\omega\tau_{\mathrm{MW}}$ for an asymmetric electrode arrangement. The DEP force is positive for low frequencies; thus, the particle is attracted to the higher electric field of the smaller electrode, and negative for higher frequencies such that the particle is repelled by the smaller electrode.

stated in the above equation the DEP force scales quadratic with the gradient of the applied electric field and particles move to regions of higher electric fields for positive and are attracted to lower electric fields for negative F_{DEP} (as indicated by the insets in supplementary figure 10). By increasing the frequency up to the transition value ($\omega\tau_{\mathrm{MW}} = 1$) the average dielectrophoretic force acting on the particles decreases,

but is still positive; hence, less particles are attracted to the high fields in the antenna gap. Therefore, to insert exactly one gold nanoparticle one must find the right frequency which is discussed below. Note that higher order multi-polar forces between two or more particles can be neglected, because the DEP force caused by the non-uniform field dominates.

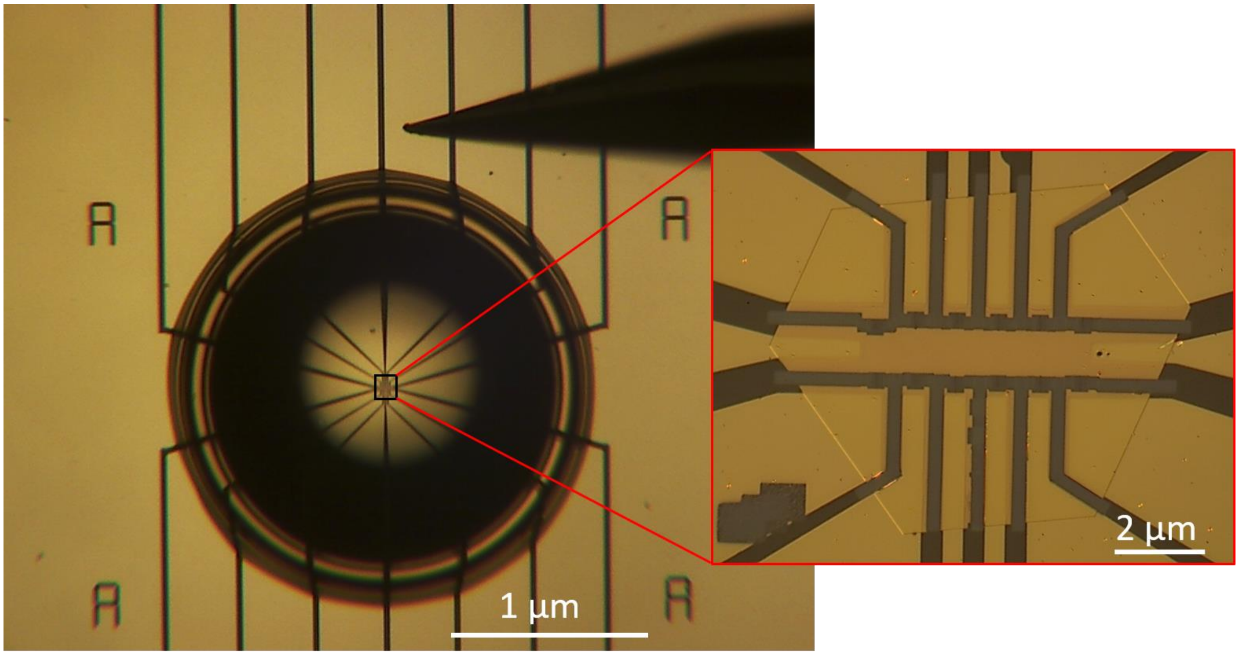

Supplementary Figure 11. Image of the DEP process on top of an inverted microscope. A 5 µl water droplet containing 30 nm gold nanoparticles is placed onto the antenna structures. The zoom-in shows the structured gold flake in the middle of the droplet. A micromanipulator is used to contact an electrode/antenna from the top while dark-field white-light scattering spectra are continuously acquired from below through the glass substrate.

The DEP is set up as depicted in supplementary figure 11. We use water-solved 30 nm gold particles (A11C-30-CTAB-1, Nanopartz, Loveland, USA) surrounded by cetyltrimethyl-ammoniumbromid (CTAB), which covers the particles and avoids contact between them and the antenna, leading to 1 nm gaps[18]. One minute of ultrasonic is applied to separate agglomerated particles, the solution is diluted with purified water 1:500 followed by an additional ultrasonic step of one minute. The gold particle solution is then dropped onto a structured gold flake and one antenna electrode is contacted via a micromanipulator that is wired to a frequency generator (DS345, Stanford Research Systems). We excite the respective antenna structure with a white-light beam and record scattering spectra to obtain a feedback in real-time (see Fig. 2a in the main text). By applying an AC electric field to the antenna structure, particles are attracted to the gap, and as soon as one or more particles enter the gap, the antenna resonance redshifts. In supplementary figure 12 the impact of a particle on the antenna spectrum is illustrated using BEM simulations and experimental results. We use the redshift as a trigger for a successful deposition and immediately switch the frequency generator off to prevent further particle depositions. Afterwards, the focus and micromanipulator are moved to the next antenna structure and the procedure is repeated. To avoid drying-up, we added water from time to time and after the DEP was finished, the droplet is rinsed away with purified water and ethanol.

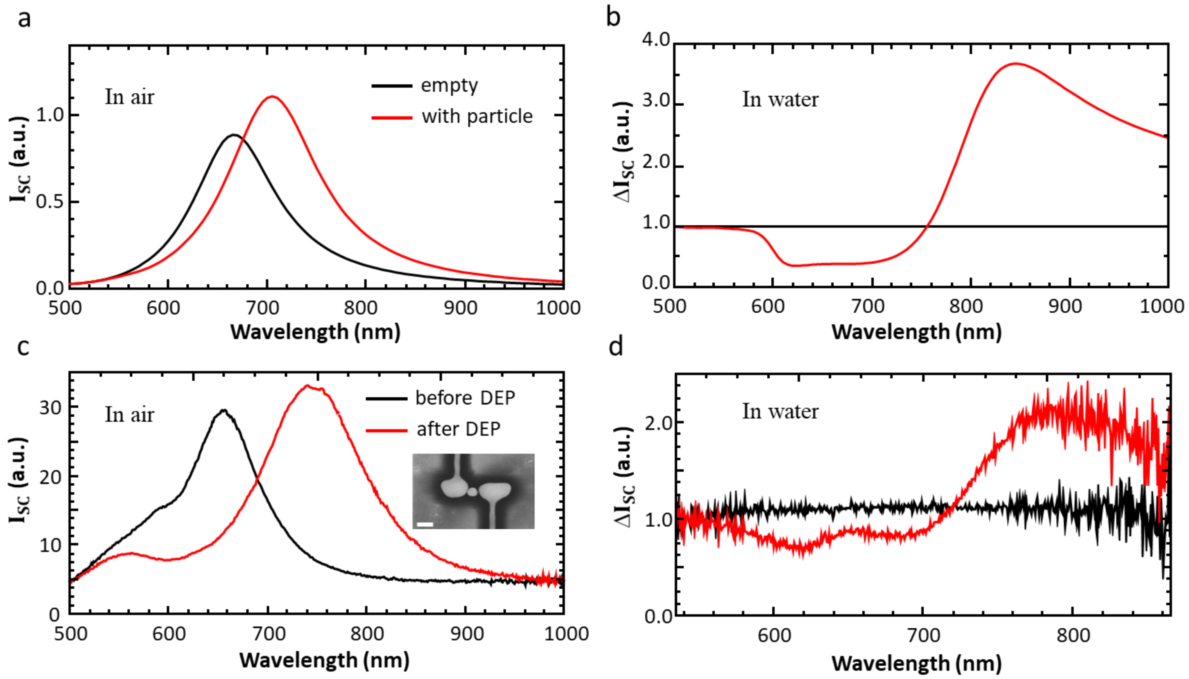

Supplementary Figure 12. Spectral response on particle deposition. (a) BEM simulated scattering spectra (I_{SC}) of a two-arm antenna without (black) and with (red) particle in air and (b) differential scattering spectra (ΔI_{SC}) of the same antenna in an aqueous environment. (c) Experimental white-light dark-field scattering spectra of an antenna before (black) and after (red) DEP in air. The inset shows that a single particle was deposited (scale bar 50 nm). (d) Differential scattering spectra acquired live during DEP before (black) and after (red) the particle was deposited.

One remaining issue of DEP is that the number of particles attracted to the gap cannot be monitored. A single particle is enough to enable light generation and any further object close to the structure just reduces its performance. Furthermore, high-resolution SEM imaging (Zeiss Ultra Plus, 10 kV, 15 µm) of optical antennas loaded with nanoparticles can only be performed after all electroluminescence and optical measurements are conducted as the electron beam locally induces high currents which breaks the tunneling barrier. To gain a high certainty for single-particle deposition the applied frequency and voltage as well as the dilution of the particle solution had to be optimized.

After a rough estimation of a gold particle concentration using drop tests we performed a DEP series with conventional dipolar nanoantennas for varying frequencies. For each frequency we applied a low starting voltage for one minute and increased it stepwise until a change in the scattering spectrum occurred. Afterwards, SEM images were acquired which are displayed in supplementary figure 13 and show that for low frequencies of 100 kHz to 1 MHz (a-c) a bunch of particles had been drawn to the structure, which can be assigned to a high F_{DEP}. As the frequency increases the average dielectrophoretic force acting on the spheres decreases and fewer particles are attracted. Single particles deposition into the antenna gap was achieved for a broad range of frequencies ranging from (d) 4 MHz to (e) 8.5 MHz. At 10 MHz (and also for higher frequencies) no particle deposition was observed anymore which can be explained by either a negative DEP (see supplementary figure 10) or by 5 V being too small to result in enough attraction.

Note, as a voltage of 1 V already leads to field strength of ~1 GeV/m in the case of a tunnel gap, we chose a maximum voltage of 5 V to avoid structural damage to the antenna once a particle is deposited. Furthermore, for increasing frequencies higher voltages are required because F_{DEP} becomes weaker but also because high-frequency losses result into lower actual voltages over the antenna gap.

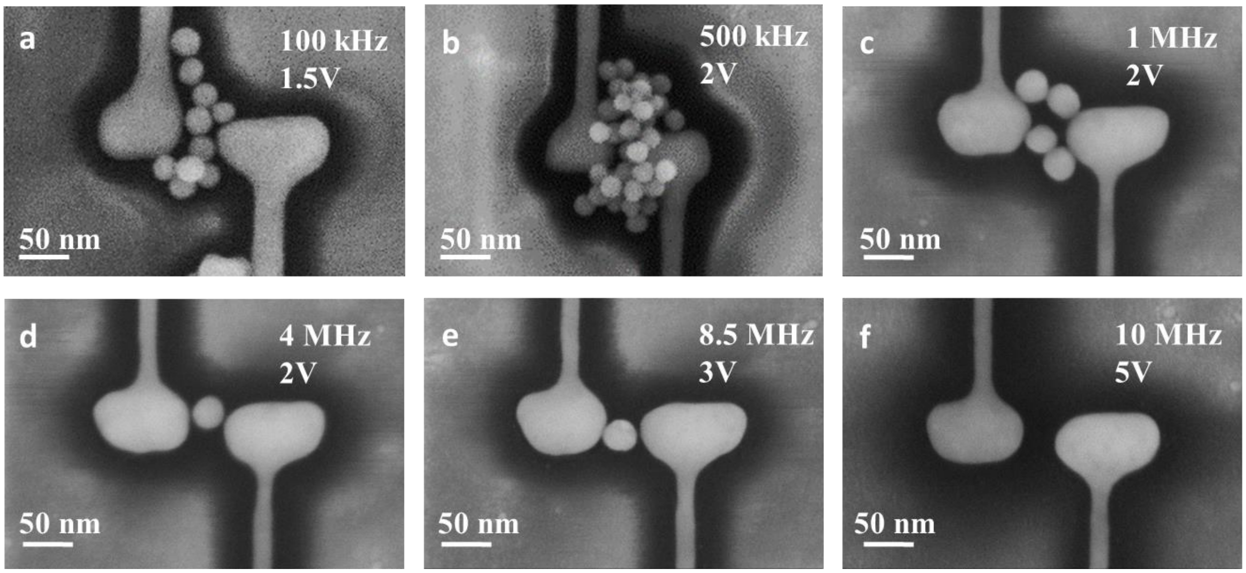

Supplementary Figure 13. Optimizing the DEP parameters using conventional dipolar nanoantennas. The concentration of the nanoparticle solution was kept constant, the frequency altered from 100 kHz to 10 MHz while each time the voltage ramped up until a spectral change was observed. Subfigures (a-f) show that for low frequencies agglomerations of particles are deposited which are reduced in particle number with increasing frequencies. Single-particle particle deposition was achieved for 4 MHz and 8.5 MHz while for 10 MHz and higher frequencies no particle deposition was observed anymore.

For each antenna geometry (dipole, feed and Yagi-Uda antennas) we had to apply slightly different parameters during DEP to attract single particles to the antenna gap. After optimal settings were found we performed DEP at 15 dipole, 12 feed and 39 Yagi-Uda antennas and the results are shown in supplementary figure 14. The success rate (one particle inside the gap) for dipole and feed antennas was 56 % and dropped slightly to 44 % for the more complex Yagi-Uda antenna. On average for all antennas this was with 48.9 % the most common event. Even though we implemented a feedback that allowed us to stepwise increasing the voltage until a change in the scattering spectrum was observed, i.e. a particle was attracted to the gap, 15 structures remained empty after reaching the voltage limit of 5 V (and waiting for 60 s). This incident was more favorable than attracting multiple particles, because empty antennas could be reused and filled in an additional DEP experiment with a new particle solution. Antennas which were loaded with several particles could already be identified in-situ by white-light scattering measurements – they featured very broad and stronger redshifted antenna resonances (see Fig 2b and e in the main text) – and were subsequently sorted out.

The placement of single particles inside the gaps results almost always in light emission based on inelastic electron tunneling. We recorded reasonable electroluminescence spectra in 78 % of these cases.

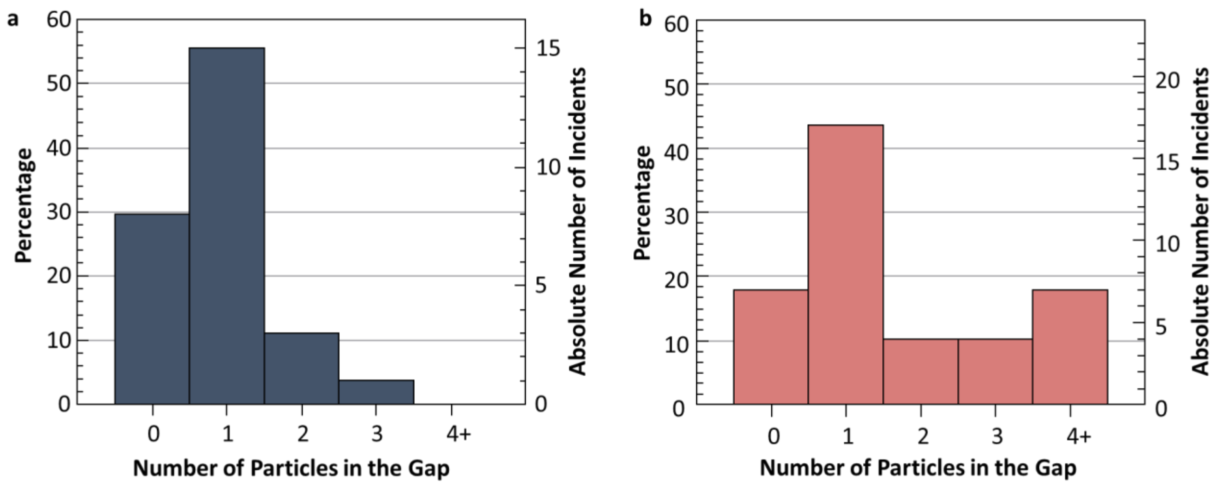

Supplementary Figure 14. Number of incidents for zero, one, two, three or more particles inside the gap of (a) 27 fabricated dipole/feed antennas and (b) 39 Yagi-Uda antennas. Overall the likelihood of having a single particle inside the gap was above 48 % and multiple particles below 29 %. Antennas without particles were reused.

Supplementary Note 10: Scalability of Fabrication

In the RF regime the directionality of Yagi-Uda antennas is increased by simply adding more directors. Optical Yagi-Uda antennas are expected to work the same; however, we first have to show that adding directors is also so simple in the case of nanostructures. Therefore, the scalability of our focused-ion beam milling technique was tested by fabricating antennas with 5, 7, 9, 11, 13 and 15 directors. The geometric parameters were kept the same and the results are depicted in supplementary figure 15. One can see that the Yagi-Uda antennas scale beautifully up to even 15 directors which demonstrates the capabilities of our advanced FIB milling technique.

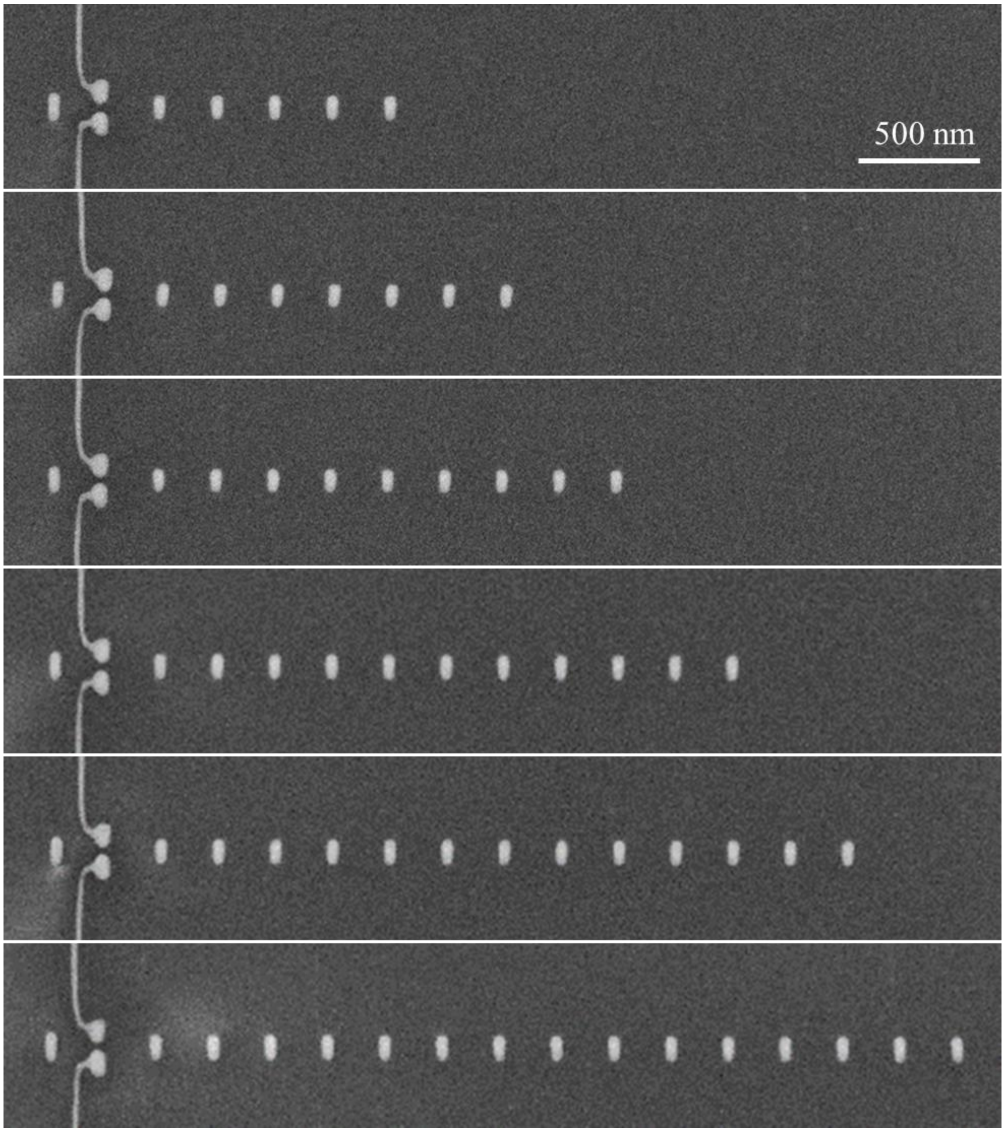

Supplementary Figure 15. Fabricated optical Yagi-Uda antennas with 5, 7, 9, 11, 13 and 15 directors. The geometric parameters are the same for all structures; only the number of directors is changed.

The next task is to show that single-particle deposition using DEP still works with large numbers of directors and that a tunnel gap is created. The optimal DEP parameters vary slightly due to the increased number of passive elements but the single-particle deposition is still successfully possible as shown in supplementary figure 16a for two exemplary antennas featuring 13 and 15 directors.

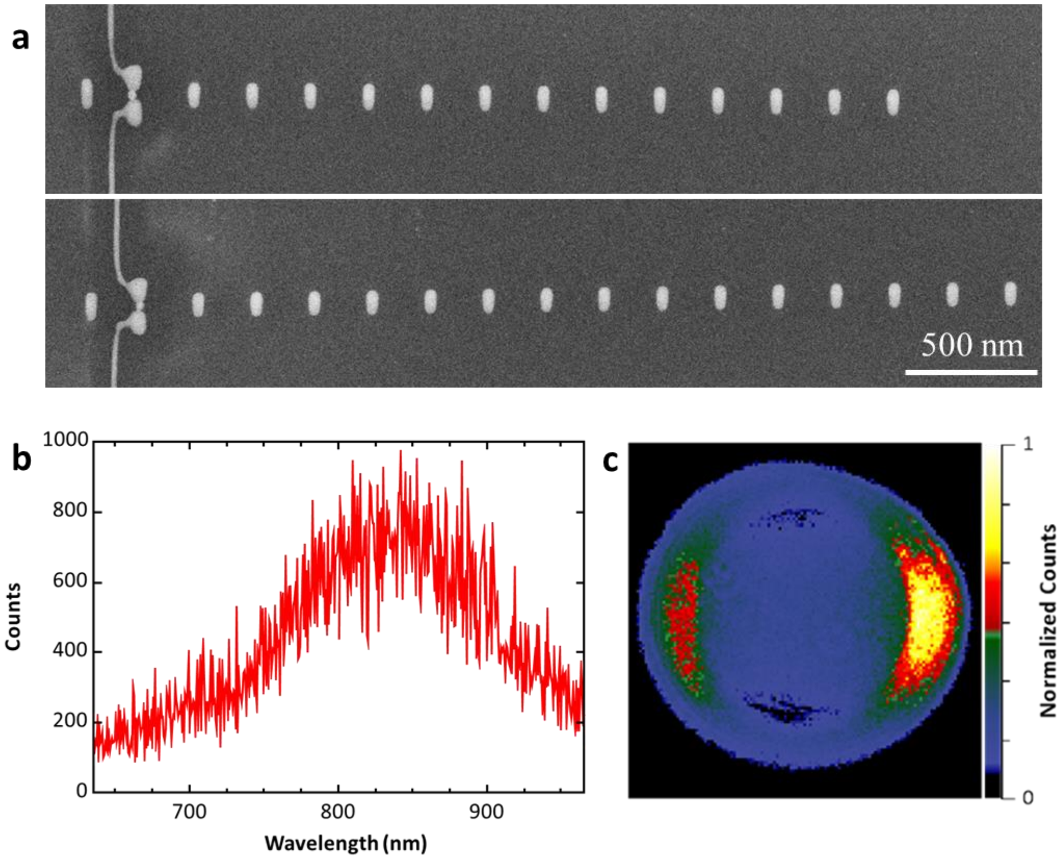

Supplementary Figure 16. (a) Yagi-Uda antennas with 13 and 15 directors after DEP showing one particle in the gap. (b) Electroluminescence spectrum and (c) emission pattern of the top antenna in (a). The structure has a FB ratio of 2.2 dB (areal method, 3.1 dB with pixel method).

IV measurements showed that a tunnel gap is formed and the electroluminescence is proven by acquiring spectra (supplementary figure 16b) as well as emission pattern (supplementary figure 16c) upon voltage application. The emission pattern is used to calculate the FB ratio yielding 2.2 dB and 3.1 dB with the "areal" and "pixel" method, respectively. This value is lower than the FB ratios measured for Yagi-Uda antennas with three directors which is discussed in detail in the main text. Nevertheless, our fabrication procedure is capable of producing optical Yagi-Uda antennas with an arbitrary number of directors that promise an outstanding directivity upon index matching (see main text).

Supplementary Note 11: Simulations

In order to better understand the low directionalities of the measured Yagi-Uda antennas with large number of directors we conducted FDTD simulations. In supplementary figure 17 near-field intensity maps for 15-director Yagi-Uda antennas on an air-glass interface as well as in an index-matched oil-glass environment (n = 1.52) are presented. The antennas are excited via a dipole source situated inside the feed gap and the length of the feed element is adapted to mimic the redshift of the gold nanoparticle in experiments. For the air-glass interface the directors further away from the feed show very little intensity because most of the light is refracted into the higher index glass substrate. Hence, directors further away do not contribute to the antenna performance, i.e. they do not increase the antenna directionality. In the case of a homogeneous surrounding the situation changes completely: No light is refracted out of the antenna plane such that all directors show significant field intensity and, hence, can contribute to a more directed emission. This is analogous to Yagi-Uda antenna in the RF regime where the antennas are also situated in a homogeneous surrounding, i.e. in air.

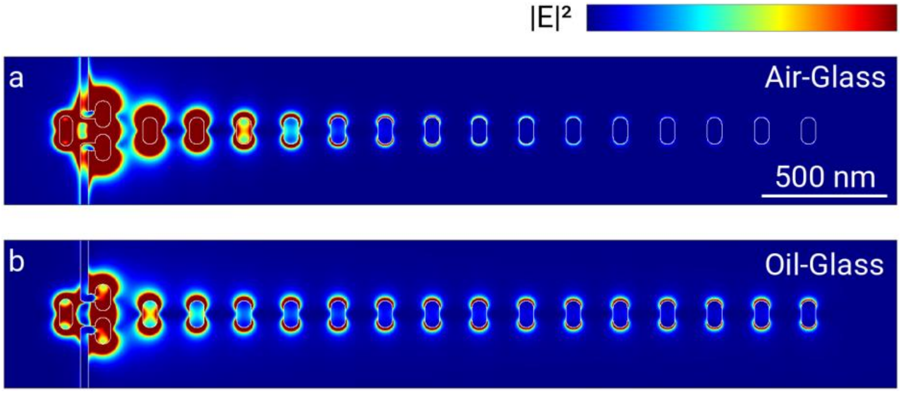

Supplementary Figure 17. FDTD calculated near-field intensity distribution of a 15-director Yagi-Uda antenna. (a) For an air-glass interface the directors further away from the feed see less and less field and, hence, contribute little to the antenna performance. (b) If the antenna is embedded in a homogeneous refractive surrounding, all directors are reached by the field and can contribute to a more directed emission. Note the intensity is scaled linearly.

For this reason, the dependence of the directionality on the number of directors is also performed in a homogeneous surrounding of n = 1.52. As a figure of merit we use the antenna gain which is the standard measure in antenna technology and it indicates how much the emitted power is enhanced in the forward direction compared to a hypothetical isotropic emitter.

A dipole in the feed-gap with polarization parallel to the long axis of the feed arms serves as source. We first calculate the logarithm of the ratio of the intensity emitted in forward direction by a Yagi-Uda antenna and the maximum intensity emitted by the bare feed-element without connector wires. This ratio in dBd (decibels-dipole) represents the forward gain over a dipolar emitter as the isolated feed element already shows a dipolar far-field emission. To obtain the gain over an isotropic emitter in dBi (decibels-isotropic) the inherent directivity of a dipolar emitter of $10 \cdot \log_{10}(1.5) \approx 1.76\ \mathrm{dBi}$ has to be added to the dBd values from the FDTD simulations.

The calculations were conducted for the structures discussed in the main text (gold + silver at 870 nm) but also for gold structures with reduced losses which was obtained by increasing the wavelength to the telecom regime (1560 nm) or by reducing the imaginary part of the gold dielectric function to 10 % of its actual values. In supplementary figure 18 the results are plotted and compared to a gold Yagi-Uda antenna (emission wavelength 870 nm) as well as a RF Yagi-Uda antenna (500 MHz). One can see that the gain increases from 12.0 dBi to 12.8 dBi (at telecom wavelengths) and 13.9 dBi (reduced imaginary part) which is already coming close to the RF values. This shows that the absorption losses of gold are the main reason for the reduced directionality in comparison to RF Yagi-Uda antennas which is further underpinned by the silver Yagi-Uda antenna discussed in the main text.

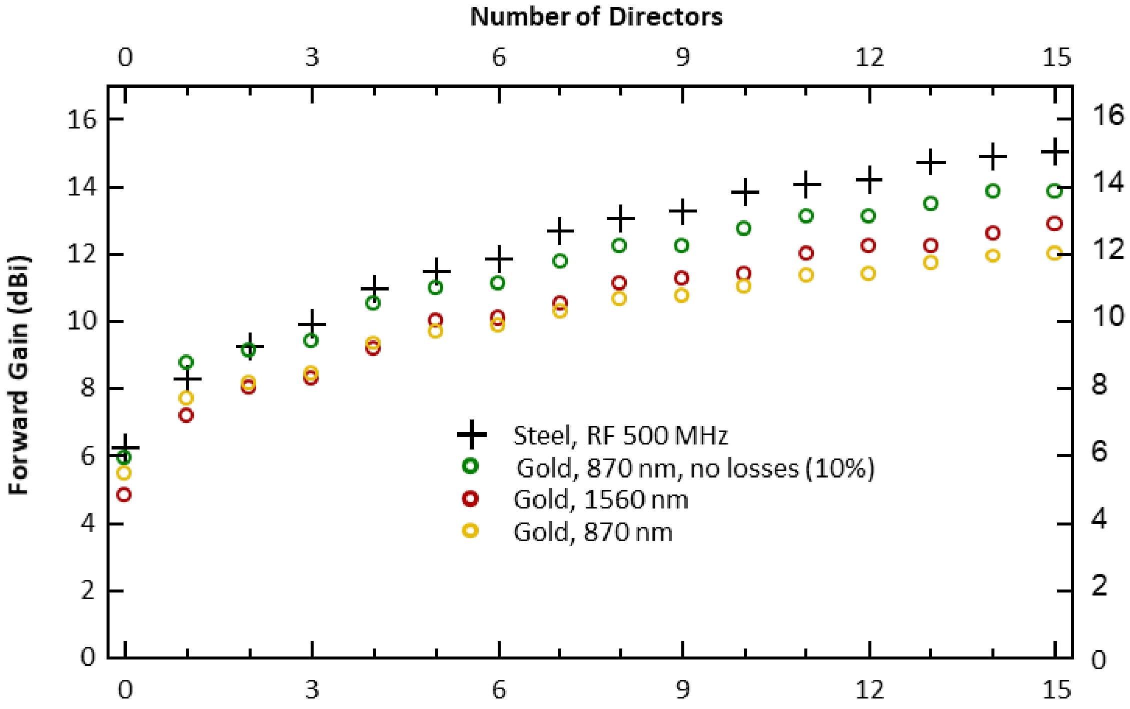

Supplementary Figure 18. Calculated forward gain as a function of the director number. The graph displays values for Yagi-Uda antennas in the RF regime (500 MHz) as well as gold Yagi-Uda antennas in the optical regime including losses (gold circles, see main text), 10% losses (green circles) and at telecom wavelengths (red circles).

The gain of the radiofrequency (RF) antenna shown in Fig. 4 of the main text and supplementary figure 18 was modeled with the publicly available NEC-2 code that is based on the method of moments to solve the integral electromagnetic equations[19,20]. It consists of a $\lambda/2$ feed element (length 0.3 m) driven by a voltage source, one reflector (length 0.302 m, spacing 0.1 m) and the directors (length 0.209 m, spacing 0.189 m). All cylindrically shaped antenna elements have a diameter of 8 mm and are discretized into 19 segments. The finite conductivity of stainless steel is taken into account.

Supplementary References

1. Curto, A. G. et al. Unidirectional Emission of a Quantum Dot Coupled to a Nanoantenna. Science 329, 930–933 (2010).
2. Gurunarayanan, S. P. et al. Electrically Driven Unidirectional Optical Nanoantennas. Nano Lett. 17, 7433–7439 (2017).
3. Hofmann, H. F., Kosako, T. & Kadoya, Y. Design parameters for a nano-optical Yagi–Uda antenna. New J. Phys. 9, 217 (2007).
4. Kosako, T., Kadoya, Y. & Hofmann, H. F. Directional control of light by a nano-optical Yagi–Uda antenna. Nat. Photonics 4, 312–315 (2010).
5. Bohren, C. F. & Huffman, D. R. Absorption and Scattering of Light by Small Particles. (John Wiley & Sons, 2008).
6. Hohenester, U. & Trügler, A. MNPBEM – A Matlab toolbox for the simulation of plasmonic nanoparticles. Comput. Phys. Commun. 183, 370–381 (2012).
7. Hohenester, U. Simulating electron energy loss spectroscopy with the MNPBEM toolbox. Comput. Phys. Commun. 185, 1177–1187 (2014).
8. Waxenegger, J., Trügler, A. & Hohenester, U. Plasmonics simulations with the MNPBEM toolbox: Consideration of substrates and layer structures. Comput. Phys. Commun. 193, 138–150 (2015).
9. Olmon, R. L. et al. Optical dielectric function of gold. Phys. Rev. B 86, 3915–3919 (2012).
10. Taflove, A. & Hagness, S. C. Computational Electrodynamics: The Finite-Difference Time-Domain Method. (2000).
11. Gedney, S. D. An anisotropic perfectly matched layer-absorbing medium for the truncation of FDTD lattices. IEEE Trans. Antennas Propag. 44, 1630–1639 (1996).
12. Kern, J. et al. Electrically driven optical antennas. Nat. Photonics 9, 582–586 (2015).
13. Prangsma, J. C. et al. Electrically Connected Resonant Optical Antennas. Nano Lett. 12, 3915–3919 (2012).
14. Krauss, E. et al. Controlled Growth of High-Aspect-Ratio Single-Crystalline Gold Platelets. Cryst. Growth Des. 18, 1297–1302 (2018).
15. Wu, X., Kullock, R., Krauss, E. & Hecht, B. Single-crystalline gold microplates grown on substrates by solution-phase synthesis. Cryst. Res. Technol. 50, 595–602 (2015).
16. Huang, J.-S. et al. Atomically flat single-crystalline gold nanostructures for plasmonic nanocircuitry. Nat. Commun. 1, 150 (2010).
17. Jones, T. B. Electromechanics of Particles. (Cambridge University Press, 2005).
18. Kern, J. et al. Atomic-Scale Confinement of Resonant Optical Fields. Nano Lett. 12, 5504–5509 (2012).
19. Burke, G. J. & Pogio, A. J. Numerical Electromagnetics Code (NEC)-Method of Moments. A User-Oriented Computer Code for Analysis of the Electromagnetic Response of Antennas and Other Metal Structures. Part 1: Program Description-Theory. Part 2: Program Description-Code. Volume 1. Revised. (1981).
20. Molteno, T. C. A. NEC2++: An NEC-2 compatible Numerical Electromagnetics Code. (2014).
